# Proliferative Hypothalamic Neurospheres Express NPY, AGRP, POMC, CART and Orexin-A and Differentiate to Functional Neurons

**DOI:** 10.1371/journal.pone.0019745

**Published:** 2011-05-11

**Authors:** Lígia Sousa-Ferreira, Ana Rita Álvaro, Célia Aveleira, Magda Santana, Inês Brandão, Sebastian Kügler, Luís Pereira de Almeida, Cláudia Cavadas

**Affiliations:** 1 Center for Neuroscience and Cell Biology, University of Coimbra, Coimbra, Portugal; 2 Department of Biology and Environment, University of Trás-os-Montes and Alto Douro, Vila Real, Portugal; 3 Department of Neurology, Viral Vectors Laboratory, University Medicine Göttingen, Göttingen, Germany; 4 Faculty of Pharmacy, University of Coimbra, Coimbra, Portugal; Universidade Federal do Rio de Janeiro, Brazil

## Abstract

Some pathological conditions with feeding pattern alterations, including obesity and Huntington disease (HD) are associated with hypothalamic dysfunction and neuronal cell death. Additionally, the hypothalamus is a neurogenic region with the constitutive capacity to generate new cells of neuronal lineage, in adult rodents.

The aim of the present work was to evaluate the expression of feeding-related neuropeptides in hypothalamic progenitor cells and their capacity to differentiate to functional neurons which have been described to be affected by hypothalamic dysfunction.

Our study shows that hypothalamic progenitor cells from rat embryos grow as floating neurospheres and express the feeding-related neuropeptides Neuropeptide Y (NPY), Agouti-related Protein (AGRP), Pro-OpioMelanocortin (POMC), Cocaine-and-Amphetamine Responsive Transcript (CART) and Orexin-A/Hypocretin-1. Moreover the relative mRNA expression of NPY and POMC increases during the expansion of hypothalamic neurospheres in proliferative conditions.

Mature neurons were obtained from the differentiation of hypothalamic progenitor cells including NPY, AGRP, POMC, CART and Orexin-A positive neurons. Furthermore the relative mRNA expression of NPY, CART and Orexin-A increases after the differentiation of hypothalamic neurospheres. Similarly to the adult hypothalamic neurons the neurospheres-derived neurons express the glutamate transporter EAAT3. The orexigenic and anorexigenic phenotype of these neurons was identified by functional response to ghrelin and leptin hormones, respectively.

This work demonstrates the presence of appetite-related neuropeptides in hypothalamic progenitor cells and neurons obtained from the differentiation of hypothalamic neurospheres, including the neuronal phenotypes that have been described by others as being affected by hypothalamic neurodegeneration. These *in vitro* models can be used to study hypothalamic progenitor cells aiming a therapeutic intervention to mitigate feeding dysfunction that are associated with hypothalamic neurodegeneration.

## Introduction

Neurons in the hypothalamus have a critical role in the control of food intake by the orexigenic and anorexigenic actions of the neuropeptides expressed in this region. Two main neuronal populations exist in the hypothalamic arcuate nucleus (ARC): the orexigenic Neuropeptide Y (NPY)/Agouti-related Protein (AGRP) neurons and the anorexigenic Pro-OpioMelanocortin (POMC)/Cocaine-and-Amphetamine Responsive Transcript (CART) neurons. Deregulation of feeding-related neuropeptides can lead to severe phenotypes in rodent models [1], [2], [3]. Moreover, some feeding related dysfunctions are associated with hypothalamic neurodegeneration, including obesity [4], [5] and Huntington's disease [6], [7].

Huntington's disease (HD) is characterized by progressive neurodegeneration that primarily occurs in the striatum and extends to other brain regions including the hypothalamus, where severe neuronal loss (>90%) occurs in the lateral hypothalamus as assessed post-mortem [8], [9]. Moreover, HD patients often show significant weight loss after disease onset, despite the appropriate caloric intake [10], which was associated with poor disease progression prognostic [11]. The hypothalamic dysfunction found in HD patients and in HD mice models includes neuronal degeneration [12], in particular loss of Orexin-A neurons in the lateral hypothalamus (LH) [7], [13] and POMC/CART neurons in the ARC [14]. Moreover, the hypothalamic expression of feeding-related neuropeptides NPY, POMC and CART was found to be reduced in transgenic models of HD [14], [15].

In addition, some studies related hypothalamic neurodegeneration with obesity. Mice models of progressive loss of hypothalamic POMC neurons [16] or hypothalamic neurodegeneration [5] develop obesity and energy balance defects. Moreover, high-fat diet induces apoptosis of NPY/AGRP and POMC neurons and leads to reduction of synaptic inputs in hypothalamic nuclei, including ARC and LH [4].

Previous studies show that the hypothalamus is a neurogenic region with the constitutive capacity to generate new cells of neuronal lineage at low rates, in adult mice [17], including neurons important for the regulation of energy balance, such as NPY-, AGRP- or POMC-expressing neurons [18], [19]. However, the origin of newborn hypothalamic cells is controversial, since they may be originated from resident neural progenitor cells in the hypothalamus or from non-resident cells that migrate to hypothalamus from other neurogenic regions [20], [21].

Recently, it was suggested that neurodegeneration stimulates hypothalamic cell proliferation in an adult mice model of progressive loss of hypothalamic AGRP neurons [19]. In opposition, blockade of cell proliferation results in decreased food intake and body adiposity in these mutant mice but not in controls [19]. For these reasons, *de novo* neurogenesis in adult hypothalamus may act as compensatory mechanism to regulate energy balance [19].

In this context, the development and characterization of *in vitro* models to study hypothalamic progenitor cells and hypothalamic neurogenesis is of utmost importance. The aim of the present work was to evaluate the expression of feeding-related neuropeptides in hypothalamic progenitor cells and their capacity to differentiate to functional neurons including those described to be affected by hypothalamic dysfunction.

Our results demonstrate the presence of appetite-related neuropeptides NPY, AGRP, POMC, CART and Orexin-A/Hypocretin-1 in hypothalamic progenitor cells and neurons obtained from the differentiation of hypothalamic neurospheres. Since these neuronal phenotypes have been described to be affected by hypothalamic neurodegeneration, our work opens new perspectives in the study of hypothalamic neurogenesis aiming a therapeutic intervention to mitigate feeding dysfunctions associated with hypothalamic neurodegeneration, as arise in Huntington disease and obesity.

## Results

### Hypothalamic neurospheres express multi-potency, astrocyte and neuronal markers

**Figure 1:**
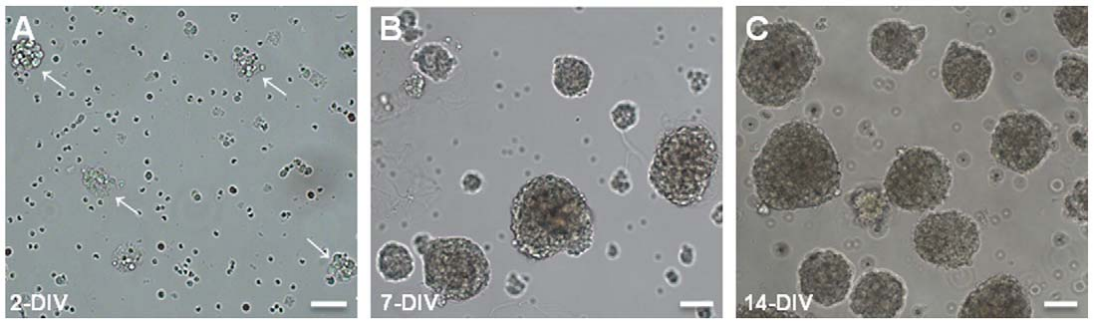
Morphology of hypothalamic neurospheres. Phase-contrast images of hypothalamic neurospheres generated from embryonic rat hypothalamic cells and cultured with growth factors in non-adhesive conditions. (**A**) Cell clusters (arrows) observed after 2-DIV, and (**B**) neurospheres observed after 7-DIV. Hypothalamic neurospheres can be maintained in undifferentiated proliferative state for at least 14 days (including 1 passage after 7-DIV) (**C**). Scale bar: 50 µm. DIV, days *in vitro*.

To investigate the proliferative potential of embryonic hypothalamic cells and evaluate the expression of feeding-related neuropeptides, we generated neurospheres from embryonic rat hypothalamic cells. After 2 days in culture, we observed small cell clusters that originate floating neurospheres after 7-DIV (Figure 1-A and 1-B). Moreover, these floating neurospheres can be maintained in undifferentiated proliferative state for at least 14-DIV (including 1 passage after 7-DIV) by culture in medium with growth factors (Figure 1-C).

**Figure 2:**
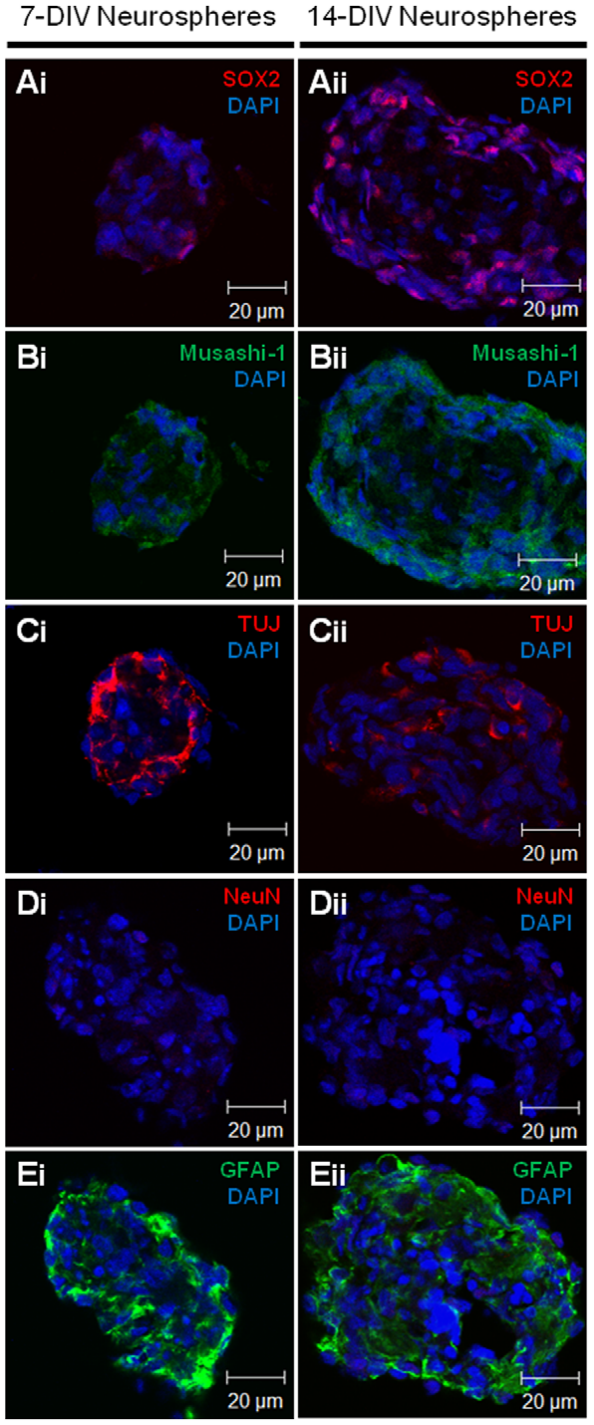
Hypothalamic neurospheres express multi-potency, neuronal and astrocytes markers. Immuno-characterization of floating hypothalamic neurospheres after 7-DIV and 14-DIV in culture with growing factors. Images of hypothalamic neurospheres expressing multi-potency marker SOX2 **(Ai and Aii)**; neural progenitor marker Musashi-1 (Bi and Bii); neuronal marker β-III-Tubulin (TUJ) (Ci and Cii) but not mature neuronal marker NeuN (Di and Dii). Hypothalamic neurospheres are also positive for astrocytes marker GFAP (Ei and Eii). DAPI, nuclear staining. DIV, days *in vitro*.

To characterize the type of cells (neuronal *versus* glial) that constitute the neurospheres we performed immunocytochemistry on 7-DIV and 14-DIV hypothalamic neurospheres (Figure 2). Hypothalamic neurospheres showed positive immunoreactivity for the SOX-2 marker confirming a multi-potent profile (Figure 2-A). Immunoreactivity for the neural progenitor marker Musashi-1 (Figure 2-B) and neuron specific marker β-III-Tubulin (TUJ) (Figure 2-C) was observed both in 7 and 14-DIV neurospheres indicating the presence of a neuronal cell lineage in the neurospheres. Conversely, differentiated neuronal nuclei (Neu-N) were not detected (Figure 2-D) confirming that the neuronal precursors present in the neurospheres are at the immature state. Moreover, strong GFAP immunoreactivity was detected both in 7 and 14-DIV neurospheres (Figure 2-E) indicating the presence of astrocytes and progenitor neural cells [22].

### Hypothalamic neurospheres express appetite-related neuropeptides: NPY, AGRP, POMC, CART and Orexin-A

**Figure 3:**
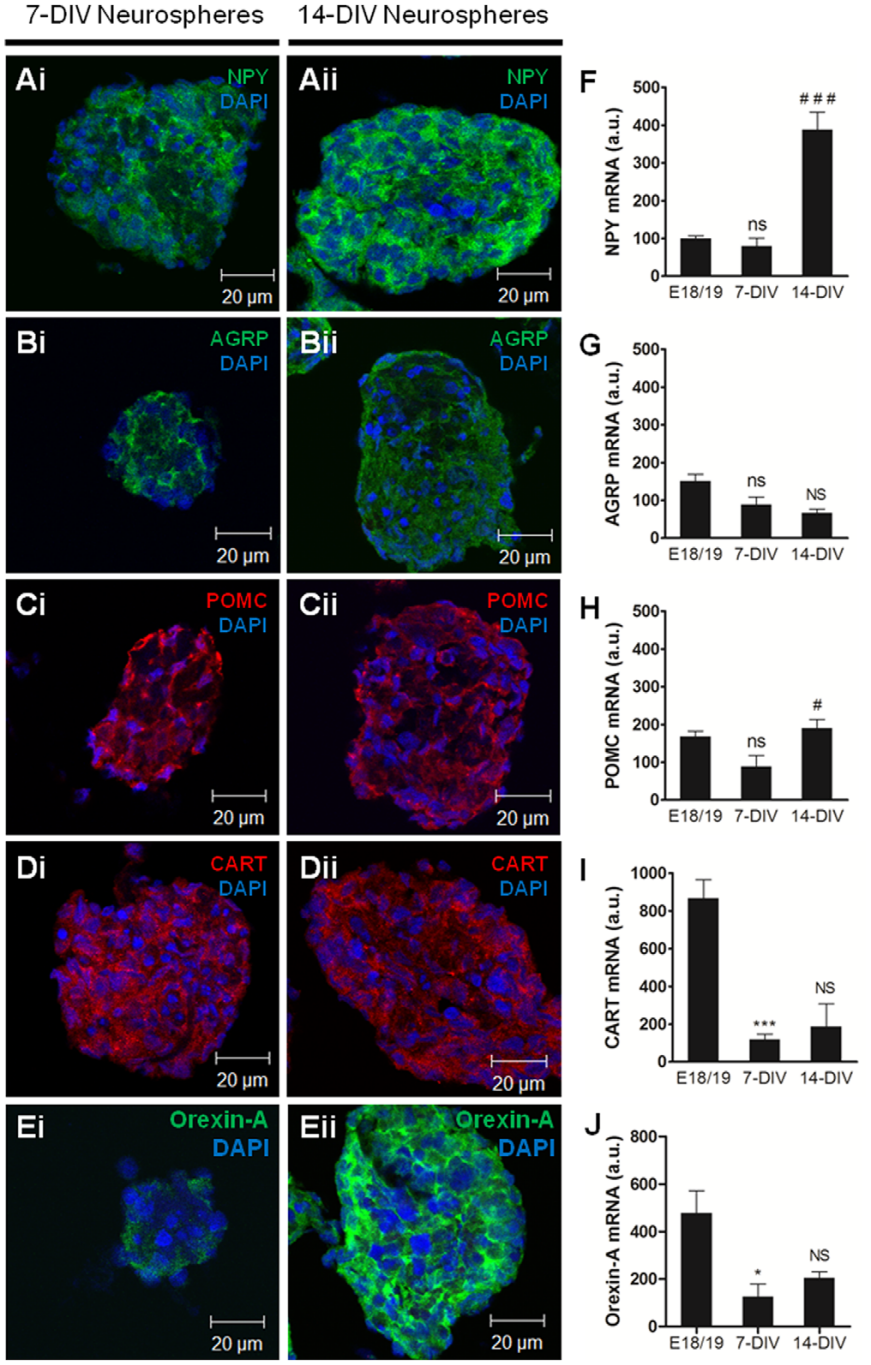
Hypothalamic neurospheres express appetite-related neuropeptides NPY, AGRP, POMC, CART and Orexin-A. Hypothalamic neurospheres were cultured for 7 days (7-DIV) or 14 days (14-DIV) in proliferative conditions. (A–E) Immunostaining of hypothalamic neurospheres sections; (F–J) relative mRNA expression of appetite-related neuropeptides in hypothalamic neurospheres. Hypothalamic neurospheres express NPY (Ai and Aii), AGRP (Bi and Bii), POMC (Ci and Cii), CART (Di and Dii) and Orexin-A (Ei and Eii). NPY immunoreactivity (Aii) and mRNA (F) increase in hypothalamic neurospheres after 14 days in proliferative conditions while AGRP mRNA (G) does not change after the same period of time. POMC mRNA increases in hypothalamic neurospheres after 14 days in proliferative conditions (H). CART mRNA (I) and Orexin-A mRNA (J) decrease after 7 days in proliferative conditions. Mean ± SEM. ns p>0.05, * p<0.05 and *** p<0.001 compared to embryonic hypothalamic cells E18/19; NS p>0.05, # p<0.01 and ### p<0.001 compared to 7-DIV hypothalamic neurospheres. n = 4/6; One-Way ANOVA. DAPI, nuclear staining. DIV, days *in vitro*.

Appetite-related neuropeptides are expressed in the hypothalamus of rat embryos at E18 [23]. Therefore, to investigate the presence of NPY, AGRP, POMC, CART and Orexin-A in hypothalamic neurospheres and evaluate their expression during culture in proliferative conditions, we evaluated the immunoreactivity and mRNA content of these neuropeptides by immunostaining and qRT-PCR, respectively (Figure 3). Hypothalamic neurospheres showed a robust immunoreactivity for NPY after 7-DIV (Figure 3-Ai) that was stronger after 14-DIV (Figure 3-Aii). The increase of NPY content was further confirmed by the 4-fold higher expression of NPY mRNA in 14-DIV neurospheres when compared to embryonic hypothalamic cells (388.44±92.42 a.u. and 99.56±14.00 a.u., respectively) (Figure 3-F).

AGRP immunostaining was also observed in 7-DIV and 14-DIV neurospheres (Figure 3-Bi and Bii) and the expression of AGRP was confirmed by qRT-PCR (Figure 3-G). Nevertheless, no differences were observed in AGRP expression in 7-DIV and 14-DIV neurospheres compared to embryonic hypothalamic cells (88.27±35.69 a.u., 66.50±15.90 a.u. and 150.82±29.24 a.u., respectively), showing that, unlike NPY, the content of AGRP in hypothalamic neurospheres is unchanged in these proliferative conditions.

Hypothalamic embryonic neurospheres also express anorexigenic POMC as shown by the POMC immunoreactivity detected in 7-DIV and 14-DIV neurospheres (Figure 3-Ci and Cii) and the presence of POMC mRNA (Figure 3-H). Moreover, the relative expression of POMC in neurospheres was increased in 14-DIV compared to 7-DIV neurospheres (189.79±42.26 a.u. and 89.02±48.34 a.u., respectively). CART immunostaining was observed both in 7-DIV and 14-DIV neurospheres (Figure 3-Di and Dii) and the expression of CART was confirmed by qRT-PCR (Figure 3-I). Nevertheless, the relative expression of CART was 7-fold lower in 7-DIV and 14-DIV neurospheres as compared to embryonic cells isolated from the hypothalamus (117.46±47.25 a.u., 187.20±176.98 a.u. and 867.04±176.67 a.u., respectively), suggesting that the content of CART in hypothalamic neurospheres decreases in proliferative conditions.

Furthermore, Orexin-A immunoreactivity was present in 7-DIV and 14-DIV hypothalamic neurospheres (Figure 3-Ei and 3-Eii, respectively). However, the levels of Orexin-A mRNA decreased after 7-DIV and 14-DIV in culture with proliferative conditions as compared to the mRNA levels of embryonic tissue from where the neurospheres were obtained (124.9±74.9 a.u., 205.5±38.1 a.u. and 476.7±131.9 a.u., respectively) (Figure 3-J).

We also isolated neurospheres from adult hypothalami (Supporting Information S1). These neurospheres (Figure S1), showed positive immunoreactivity for NPY, AGRP, POMC and CART (Figure S2). All together, these results show that hypothalamic adult and embryonic neurospheres express feeding-related peptides and that their expression is modified during culture in proliferative conditions.

### Hypothalamic neurospheres cells differentiate into mature neurons and glial cells

**Figure 4:**
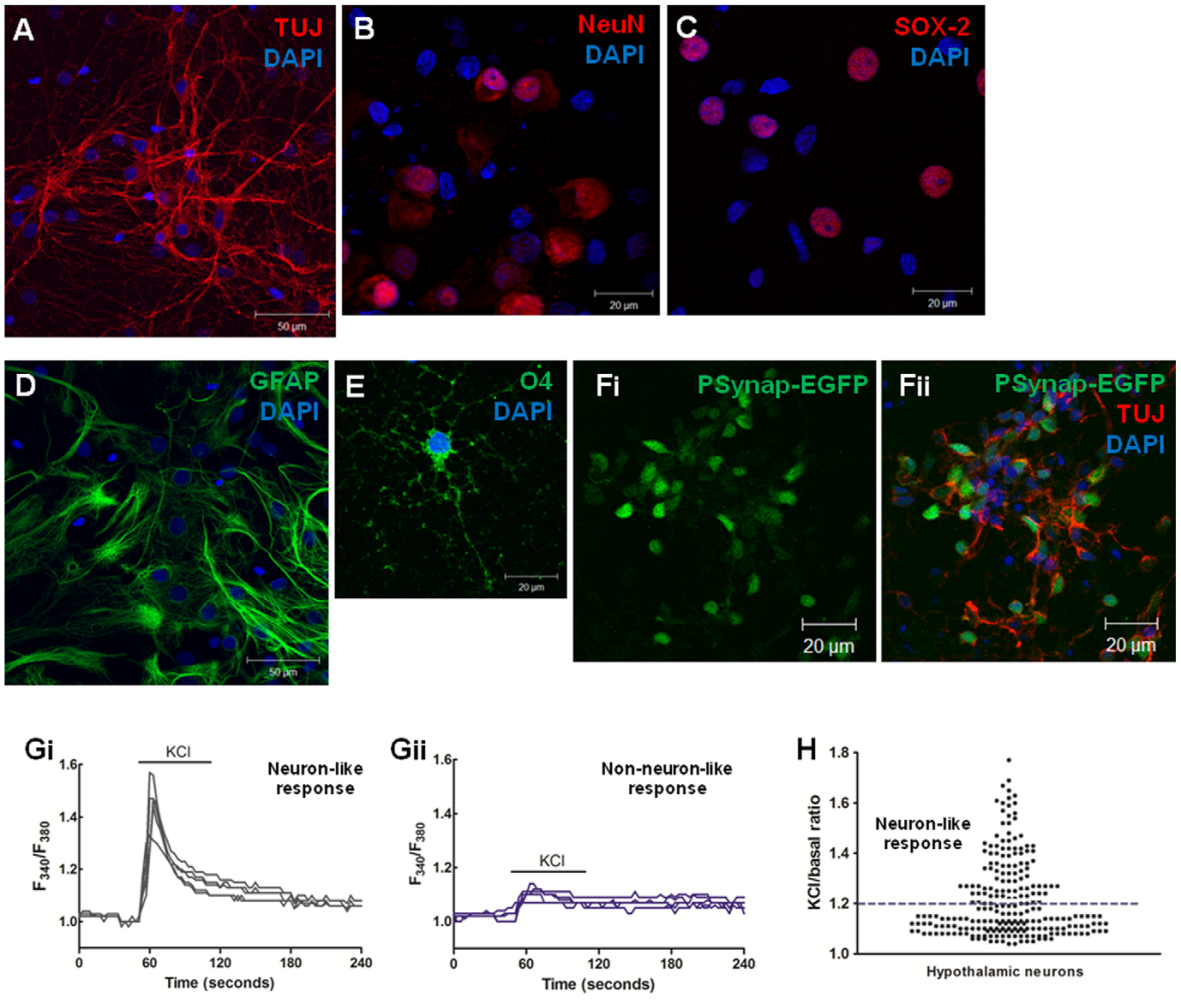
Hypothalamic neurospheres differentiate to mature functional neurons. Characterization of hypothalamic neurons culture obtained from plated neurospheres after 18 days in differentiation conditions. Differentiated hypothalamic cultures predominantly express neuronal marker TUJ (A) and mature neurons marker NeuN (B) but also multi-potency marker SOX-2 (C). Hypothalamic cultures also express astrocytes marker GFAP (D) and oligodendrocytes marker O4 (E). (F) Infection of hypothalamic neurons culture with AAV vectors expressing EGFP under the control of the synapsin neuronal specific promoter (Psynap), after 10 days of differentiation. (Fi) Neurons expressing the EGFP protein showing that the neuronal promoter is active and (Fii) merge image with neuronal marker TUJ. DAPI, nuclear staining. Representative recordings of intracellular calcium concentrations ([Ca^2+^]_i_) of hypothalamic cells with neuron-like response (Gi) or non-neuron-like response (Gii) to 50 mM KCl depolarization. (H) Distribution of hypothalamic cells according to the KCl/basal ratio obtained after a potassium stimulus. n = 3 independent culture preparations. AAV, adeno-associated virus; EGFP, enhanced green fluorescent protein.

In order to evaluate the type of cells obtained from the differentiation of hypothalamic neurospheres, we performed immunocytochemistry of plated cells using neurons, astrocytes and oligodendrocytes markers. Hypothalamic neurospheres differentiate into neurons as shown by the robust immunostaining for neuron specific markers β-III-Tubulin and Neu-N (Figure 4-A and B, respectively). Moreover, approximately half of the total nuclei were positive for the Neu-N marker (48.3±3.7% of NeuN-positive nuclei/DAPI total nuclei), indicating that half of the cells are differentiated mature neurons. Nevertheless, some cells in the hypothalamic neurons cultures kept the expression of multi-potency markers as shown by the immunostaining for SOX-2 marker (Figure 4-C). In fact, half of the total number of cells was positive for the SOX-2 marker (53.6±0.9% of SOX-2 positive nuclei/DAPI total nuclei) (Figure 4-C). Additionally, GFAP-positive cells were detected indicating the presence of astrocytes (Figure 4-D). The presence of oligodendrocytes was demonstrated by O4 immunoreactivity (Figure 4-E), but this cell type constituted less than 1% of the culture.

To further characterize the neuronal differentiation state of the hypothalamic neuronal cultures, we evaluated the activity of the neuronal-specific synapsin promoter. This promoter mediates neuron-specific transgene expression when delivered by AAV vectors [24]. Therefore, as a proof-of-principle of the neuronal phenotype we infected 2-DIV and 10-DIV hypothalamic neurons cultures with recombinant AAV vectors expressing the EGFP protein (AAV-EGFP) and allowed the transgene expression for seven days. After the infection of 2-DIV hypothalamic neurons with AAV-EGFP we could not detect any EGFP expression (data not shown), suggesting that the neuronal-specific promoter is not yet expressed in cultures with this age. Conversely, the infection of 10-DIV hypothalamic neurons with AAV-EGFP resulted in strong expression of EGFP (Figure 4-Fi) in neurons positive for the TUJ staining (Figure 4-Fii).

### Hypothalamic neurospheres cells differentiate into functional neurons

Additionally, we tested whether the hypothalamic neurons obtained from differentiation of neurospheres cells were functional. For this purpose we recorded the intracellular calcium concentrations ([Ca^2+^]_i_) of single neurons upon 50 mM KCl depolarization, which is considered a marker of functional differentiated neurons [25], [26]. Hypothalamic neurons were divided in two groups based on the KCl/basal ratio of the [Ca^2+^]_i_ amplitudes: cells presenting a KCl/basal ratio >1.2 were considered to have an neuron-like response (Figure 4-Gi) and cells presenting a KCl/basal ratio <1.2 were considered to have an non-neuron-like response (Figure 4-Gii). Our results show that approximately 40% of the cells analyzed (42.1±8.1% of the total number of cells) had a neuron-like response to 50 mM KCl, as shown in the distribution of KCl/basal ratio of individual hypothalamic neurons (Figure 4-H). Interestingly, this percentage of mature functional neurons obtained by single-cell calcium imaging is consistent with the one obtained from immunostaining for mature neurons with the NeuN marker (described above). Therefore, these evidence confirms that hypothalamic neuronal cultures obtained from plated neurospheres can differentiate into functional neurons after culture in differentiation conditions.

### Hypothalamic neuronal cultures derived from plated neurospheres express appetite-related neuropeptides NPY, AGRP, POMC, CART and Orexin-A

To investigate the phenotype of cells resulting from the differentiation of hypothalamic neurospheres, we evaluated the expression of appetite-related neuropeptides NPY, AGRP, POMC, CART and Orexin-A.

**Figure 5:**
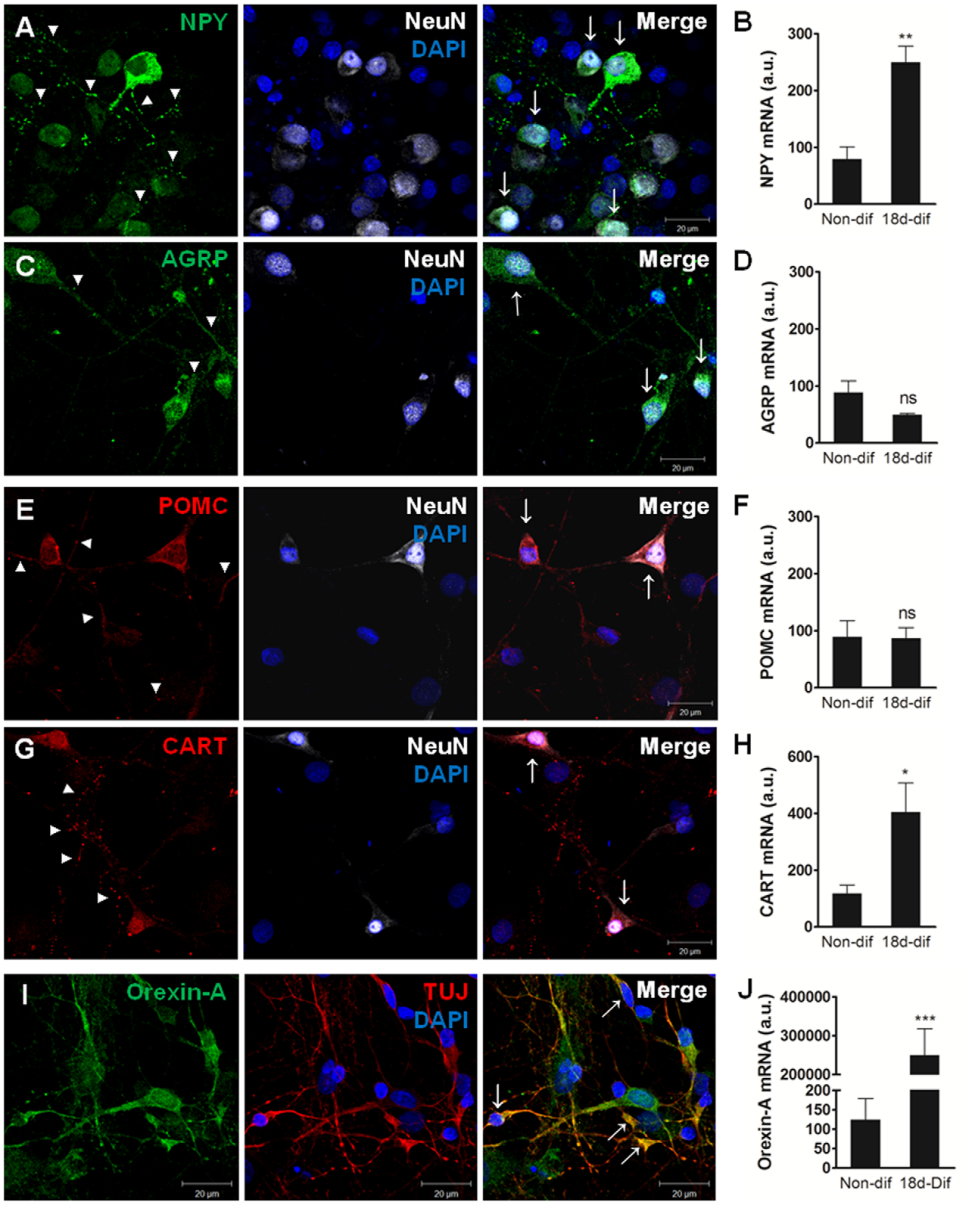
Hypothalamic neurospheres differentiate to mature neurons expressing appetite-related neuropeptides. Images of hypothalamic neurons cultures obtained from plated neurospheres after 18 days in differentiation conditions. NPY vesicular immunoreactivity (arrow-heads) (A) and NPY-positive neurons (arrows) (A-merge); AGRP vesicular immunoreactivity (arrow-heads) (C) and AGRP-positive neurons (arrows) (C-merge); POMC vesicular immunoreactivity (arrow-heads) (E) and POMC-positive neurons (arrows) (E-merge); CART vesicular immunoreactivity (arrow-heads) (G) and CART-positive neurons (arrows) (G-merge); Orexin-A immunoreactivity (I) and Orexin-A positive neurons (arrows) (I-merge). (B, D, F, H, J) Relative mRNA expression of feeding-related neuropeptides in hypothalamic neuronal cultures. The expression of NPY, CART and Orexin-A increases in differentiated hypothalamic cultures while the expression of AGRP and POMC is unchanged. Mean ± SEM. ns p>0.05, * p<0.05, ** p<0.01 and *** p<0.001, compared to non-differentiated hypothalamic neurospheres. n = 4/5; t-test. DAPI, nuclear staining; non-dif, non-differentiated hypothalamic neurospheres; 18d-dif, hypothalamic neurons culture after 18 days of differentiation.

Hypothalamic neurospheres differentiate into orexigenic neurons expressing NPY and AGRP (Figure 5-A and 5-C, respectively). Furthermore, these neurons are mature as shown by co-localization of NPY-ir and AGRP-ir with the mature neurons marker Neu-N (Figure 5-A merge and 5-C merge, respectively). Additionally, the expression of NPY mRNA increased 2-fold in differentiated hypothalamic cultures as compared to undifferentiated neurospheres from where the cultures were obtained (249.58±46.80 a.u. and 79.37±31.45 a.u, respectively) (Figure 5-B). The expression of AGRP mRNA was unchanged in differentiated hypothalamic cultures compared to undifferentiated neurospheres (49.39±4.03 a.u. and 88.27±35.69 a.u., respectively) (Figure 5-D).

Mature anorexigenic neurons expressing POMC and CART were also present in hypothalamic cultures derived from plated neurospheres (Figure 5-E and 5-G, respectively) as observed by co-localization with mature neurons marker NeuN (Figure 5-E merge and 5-G merge, respectively). The POMC mRNA expression was not significantly different between the differentiated hypothalamic neurons and the undifferentiated neurospheres from where the neurons were obtained (86.64±27.86 a.u. and 89.02±48.34 a.u., respectively) (Figure 5-F). However, CART mRNA expression was 3.5-fold higher in differentiated hypothalamic cultures than in undifferentiated neurospheres (404.01±163.40 a.u. and 117.46±47.25 a.u., respectively) (Figure 5-H), indicating an increase of the CART content in hypothalamic neurons cultured in differentiation conditions. Additionally, hypothalamic neurospheres differentiate to Orexin-A positive neurons as shown by co-localization of Orexin-A immunoreactivity and neuronal marker TUJ (Figure 5-I merge). Furthermore, there was a 2000-fold increase in the mRNA expression of Orexin-A in the differentiated neuronal cultures compared to the hypothalamic neurospheres (250194.4±104396.5 a.u. and 124.9±74.9 a.u., respectively) (Figure 5-J). These results show that hypothalamic neurospheres can differentiate to a neuronal culture constituted by mature orexigenic and anorexigenic neurons.

### Hypothalamic neurospheres-derived neurons express glutamate transporter EAAT3

**Figure 6:**
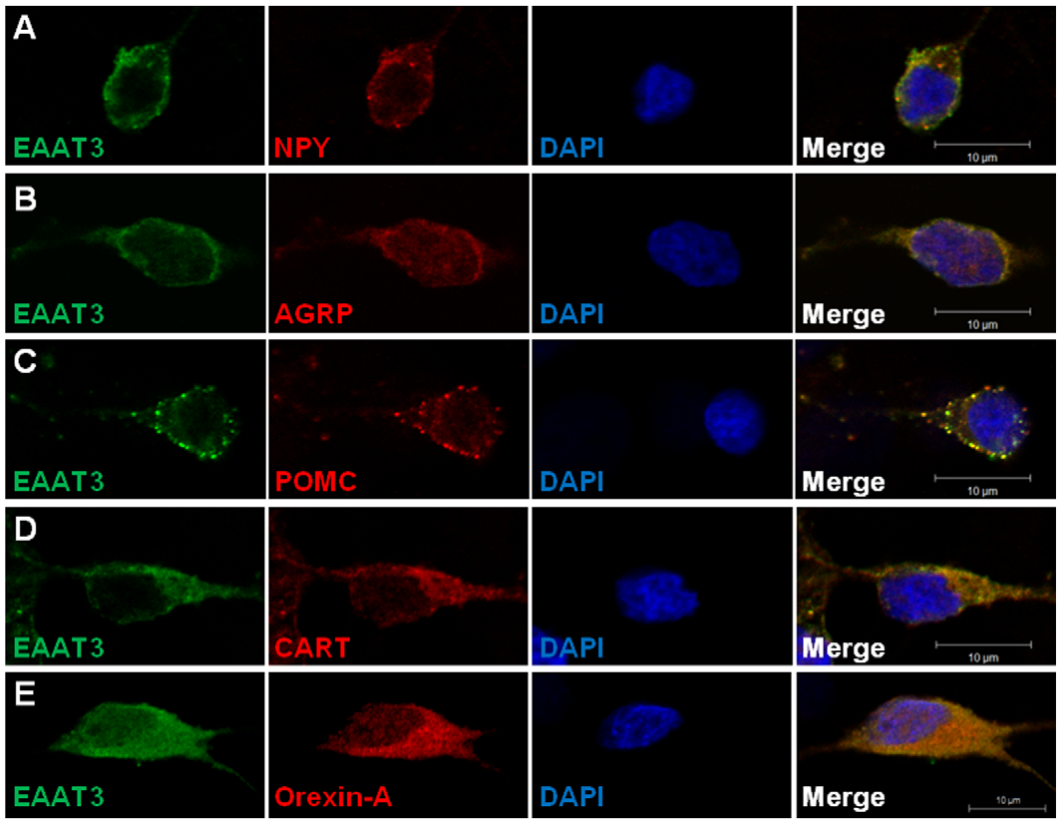
Hypothalamic neurospheres-derived neurons express glutamate transporter EAAT3. Images of hypothalamic neurons obtained from plated neurospheres after 18 days in differentiation conditions and double-labeled with appetite-related neuropeptides and glutamate transporter EAAT3. EAAT3 immunoreactivity is present in NPY- (**A**), AGRP- (**B**), POMC- (**C**), CART- (**D**) and Orexin-A positive cell bodies (**E**). DAPI, nuclear staining.

Glutamate transporter EAAT3 is expressed by adult hypothalamic neurons [27]. Therefore, we investigate the presence of the excitatory amino acid transporter 3 in NPY, AGRP, POMC, CART and Orexin-A neurons obtained from the differentiation of hypothalamic neurospheres by double immunostaining (Figure 6). EAAT3 immunoreactivity is present in the hypothalamic neurospheres-derived neurons including in NPY and AGRP positive cell bodies (Figure 6-A and B, respectively) as well as in POMC, CART and Orexin-A positive cell bodies (Figure 6-C, D, E, respectively).

### Hypothalamic neurospheres-derived neurons respond to hormones ghrelin and leptin

**Figure 7:**
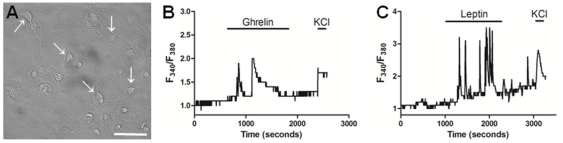
Hypothalamic neurospheres-derived neurons respond to ghrelin and leptin. (A) Phase-contrast images of hypothalamic neurons (arrows) obtained from plated neurospheres after 18 days in differentiation conditions. Representative recordings of intracellular calcium concentrations ([Ca^2+^]_i_) of hypothalamic cells responding to (B) Ghrelin (10^−10^ M) or (C) Leptin (10^−12^ M) followed by to 50 mM KCl depolarization. Scale bar: 50 µm.

Hypothalamic neurons respond to specific hormones such as ghrelin that activates the orexigenic NPY neurons [28] and leptin that activates the anorexigenic POMC neurons [29]. To investigate whether the neurons obtained from the differentiation of hypothalamic neurospheres respond to these specific stimuli, we measured the intracellular calcium concentrations ([Ca^2+^]_i_) of single neurons (Figure 7-A arrows) upon ghrelin or leptin incubation. Stimulation with ghrelin 10^−10^ M increased the [Ca^2+^]_I_ in 11 of the 89 neurons examined (12.4±1.5%) (Figure 7-B) while the stimulation with leptin 10^−12^ M increased the [Ca^2+^]_I_ in 11 of the 122 neurons examined (9.0±1.0%) (Figure 7-C). Furthermore, all the ghrelin or leptin responsive neurons had a neuron-like respond to the ending 50 mM KCl depolarization. These results indicate that the neurospheres-derived hypothalamic neurons are functionally responding to specific stimuli, and that neurons with orexigenic and anorexigenic profiles are obtained from the differentiation of hypothalamic progenitor cells.

## Discussion

In the present study we isolated neuronal precursor cells from embryonic rat hypothalamic tissue and expanded them in proliferate culture as free-floating neurospheres. These neurospheres were mainly constituted by neuronal precursor cells as shown by immunostainning for neural precursor marker Musashi-1 and neuronal immature marker TUJ. The hypothalamic precursor cells differentiated to functional neurons expressing feeding-related neuropeptides NPY, AGRP, POMC, CART and Orexin-A. In addition, the orexigenic and anorexigenic profile of the neurospheres-derived neurons was confirmed by functional response to hormones leptin and ghrelin. Furthermore, these neurons showed specific features of the adult hypothalamic neurons including neuropeptides vesicular immunoreactivity and co-localization with glutamate transporter EAAT3.

Viable hypothalamic neuronal cultures are difficult to obtain and have a short life-span [30], usually with a minimal number of healthy peptide-secreting neurons. Therefore, we used the proliferative capacity of hypothalamic neurospheres to overcome these limitations and obtain a high cellular yield culture method. The method here described constitutes a useful tool to obtain and study hypothalamic neurons, including the differentiation and the influence of drugs acting in the CNS in this process.

The hypothalamus was identified as a neurogenic region in the adult rodent brain with constitutive generation of new neuronal cells [17], [20]. However, there is a controversy in respect to the origin of newborn hypothalamic cells and whether they originate from resident neural progenitor cells in the hypothalamus or from non-resident cells that migrate to the hypothalamus from other neurogenic regions. In fact, induction of hypothalamic neurogenesis by neurotrophic factors also increases the number of new-born cells in the subventricular zone of the lateral ventricles which may be migrating from this area to the hypothalamus [20]. Nevertheless, other evidence support the role of the hypothalamus as a neurogenic niche including the presence of “pairs” of new-born cells in the hypothalamic parenchyma, showing that cell division occurs within the hypothalamus [17], [20]. More recently, it was shown that the cellular organization of the hypothalamic ventricle wall exhibit similar structural characteristic to other well known neurogenic niches in the adult brain [21], [31].

Our study reveals that appetite-related neuropeptides are expressed in hypothalamic progenitor cells, and that their levels are influenced by proliferative conditions. In fact, the expression of NPY and POMC increases during the culture of hypothalamic neurospheres with growth factors, while the expression of CART and Orexin-A strongly decreases.

We can hypothesize that the increased content of NPY in proliferative hypothalamic neurospheres is promoting the progenitor cells self-renewal since some studies showed that NPY promotes the proliferation of progenitor cells [32], although in other regions of the nervous system [33], [34], [35]. This may represent a mechanism to ensure the establishment and maintenance of feeding circuits. Conversely, the simultaneous increase of the anorexigenic neuropeptide POMC in proliferative hypothalamic neurospheres suggests a mechanism to balance anorexigenic and orexigenic feeding signals during proliferation.

Most of the new-born cells in the adult hypothalamus take the neuronal fate [17], [18], [20] and can present neuronal phenotypes implicated in the regulation of energy balance [18], [19]. Moreover, *in vitro* hypothalamic progenitor cells isolated from adult and embryonic hypothalamus can also differentiate to neuro-endocrine neurons [36], [37]. However, these studies did not show that the new hypothalamic neurons are indeed functional cells. In the present work, we showed that functional neurons can be obtained from hypothalamic progenitor cells, as tested by single cell image of intracellular Ca^2+^ concentration upon an orexigenic stimulus (Ghrelin) or an anorexigenic stimulus (Leptin).

Neurodegeneration of hypothalamic neurons is correlated to some feeding dysfunctions including obesity [4], [5] and neurodegenerative diseases, such as Huntington disease [6], [7]. Interestingly, it was recently suggested that hypothalamic proliferation can act as a compensatory mechanism of the feeding circuits in response to neurodegeneration [19].

In our study we showed that hypothalamic progenitor cells express feeding-related neuropeptides and differentiate to mature neurons including those described to be affected by hypothalamic neurodegeneration in obesity and Huntington disease: NPY, AGRP, POMC, CART and Orexin-A neurons. Furthermore, there is an increase in the NPY, CART and Orexin-A contents on hypothalamic neuronal cultures obtained from neurospheres showing that the resident progenitor cells of the hypothalamus may constitute a source of new feeding-related neurons to compensate hypothalamic neurodegeneration. Therefore, our work opens new perspectives in the study of hypothalamic neurogenesis aiming a therapeutic intervention to mitigate feeding dysfunctions associated with hypothalamic neurodegeneration.

## Materials and Methods

### Animals

Female Wistar rats were purchased from Charles River Laboratories. All experimental procedures were performed in accordance with the European Union Directive 86/609/EEC for the care and use of laboratory animals.

### Hypothalamic neurospheres

Floating hypothalamic neurospheres were obtained as previously described with modifications [30], [37]. Hypothalamic tissue was dissected from rats at embryonic days 18–19 (E18–19). Batches of 10–14 tissue fragments were successively dissociated by means of a Pasteur pipette in a PBS solution with 5.5 mM glucose (Sigma), 100 U/ml penicillin and 100 µg/ml streptomycin (both from Gibco), with no enzymatic digestion. Cells were suspended in 10 mL of DMEM-F12/Glutamax supplemented with growth factors (10 ng/mL fibroblast growth factor-2 and 10 ng/mL epidermal growth factor), 100 U/ml penicillin, 100 µg/ml streptomycin and 1% B27 supplement (all from Gibco). The floating neurospheres were allowed to growth in uncoated 25 cm^2^ flask and maintained at 37°C with 5% CO_2_. After 6/7 days of culture, neurospheres were collected by centrifugation, dissociated and kept in culture for additional 7 days by re-suspension in fresh DMEM-F12/Glutamax medium with growth factors (passage).

### Differentiation of hypothalamic neurospheres cells

After 6–7 days in culture, neurospheres were collected by centrifugation, dissociated and plated in Poly-D-Lysine coated 12-well culture plates for RNA extraction or glass cover-slips for immunocytochemistry, rAAV infection and single-cell calcium imaging, at a density of 1.0×10^7^ cells per cm^2^. To obtain differentiated hypothalamic neurons culture, the plated neurospheres were allowed to differentiate for 18 days in Neurobasal medium with 500 µM L-Glutamine, 2% B27 supplement, 100 U/ml penicillin and 100 µg/ml streptomycin (all from Gibco), with no growth factors. After 18 days in culture (18-DIV), differentiated hypothalamic neurons cultures were used for immunocytochemistry, rAAV infection and single-cell calcium imaging.

### Immunocytochemistry of hypothalamic neurospheres and differentiated hypothalamic neurons culture

OCT embedded neurospheres were cut into 8 µm sections on a cryostat (Leica) and collected to Super Frost Plus glass slides (Thermo Scientific). Hypothalamic neurospheres and differentiated hypothalamic neurons culture were fixed with 4% paraformaldehyde (Sigma) and permeabilized with 1% Triton X-100 (Sigma) followed by one hour blocking with 3% BSA (Sigma) and incubation with the primary antibody overnight at 4°C. Thereafter, the incubation with the secondary antibodies was performed for one hour at room temperature. Nuclei were stained with 4′6-diamidino-2-phenylindoline, DAPI (1∶5000, Applichem).The primary antibodies used were: mouse anti-NeuN (1∶500, Chemicon), mouse anti-beta-III-tubulin, TUJ (1∶500, Covance), rabbit anti-Glial Fibrillary Acidic Protein, GFAP (1∶400, Dako), mouse anti-oligodendrocytes marker O4 (1∶100, Millipore), mouse anti-SOX-2 (1∶200, R&D Systems), goat anti-EAAT3 (1∶500, Chemicon), rabbit anti-NPY (1∶100, Sigma), rabbit anti-AgRP (1∶100), rabbit anti-POMC (1∶100), rabbit anti-CART (1∶200) and rabbit anti-Orexin-A (1∶200) (all from Phoenix Pharmaceuticals). And the secondary antibodies used were: goat anti-mouse Alexa-Flour 594, goat anti-rabbit Alexa-Flour 488 and rabbit anti-goat Alexa-Fluor 488 (all 1∶200, Invitrogen). Fluorescence images were recorded using a confocal microscope (LSM 510 Meta; Zeiss). The percentage of Neu-N or SOX-2 positive cells was calculated from two different cover-slips in a total of eight independent microscopic fields (4 microscopic fields per cover-slip) and normalized to total number of nuclei with DAPI staining (approximately 70 nuclei per microscopic field). The procedure was performed for three independent culture preparations.

### Isolation of total RNA from hypothalamic neurospheres, differentiated hypothalamic neurons culture and embryonic tissue and cDNA synthesis

Hypothalamic neurospheres and embryonic hypothalamic tissue mechanically dissociated were collected by centrifugation. Thereafter, the medium was removed and cells were immediately frozen in dry ice. The medium of differentiated hypothalamic neurons culture was removed and cells were immediately frozen in dry ice. Samples were kept at −80°C until RNA extraction. Total RNA was extracted from 7-DIV and 14-DIV hypothalamic neurospheres, 18-DIV differentiated hypothalamic neurons culture and embryonic hypothalamic tissue using the RNeasy Mini Kit (QIAGEN) according to the manufacturer's instructions. Briefly, after cell lyses, the total RNA was adsorbed to a silica matrix, washed with the recommended buffers and eluted with 30 µl of RNase-free water by centrifugation. Total amount of RNA was quantified by optical density (OD) measurements using a ND-1000 Nanodrop Spectrophotometer (Thermo Scientific) and the purity was evaluated by measuring the ratio of OD at 260 and 280 nm. In addition, RNA quality was assessed by gel electrophoresis. Prior to cDNA conversion, samples were treated with RNase-free DNAse (QIAGEN) to eliminate any contamination with genomic DNA. cDNA was obtained from the conversion of 1 µg of total RNA using the iScript Select cDNA Synthesis Kit (Bio-Rad) according to the manufacturer's instructions, diluted 100× times in RNase-free water and stored at −20°C.

### Quantitative real time polymerase chain reaction (qRT-PCR)

Quantitative PCR was performed in an iQ5 thermocycler (Bio-Rad) using 96-well microtitre plates and the QuantiTect SYBR Green PCR Master Mix (QIAGEN). The primers for the target rat genes (NPY, NM_012614; AgRP, NM_033650; POMC, NM_139326; CART, NM_017110 and Orexin-A, NM_013179) and the reference gene (rat HPRT, NM_012583) were pre-designed and validated by QIAGEN (QuantiTect Primers, QIAGEN). A master mix was prepared for each primer set containing the appropriate volume of 2× QuantiTect SYBR Green PCR Master Mix and 10× QuantiTect Primer (both from QIAGEN). For each reaction, 18 µl of master mix were added to 2 µl of template cDNA. All reactions were performed in duplicate (two cDNA reactions per RNA sample) at a final volume of 20 µl per well. The reactions were performed according to the manufacturer's recommendations: 95°C for 15 min. followed by 40 cycles at 94°C for 15 sec, 55°C for 30 sec and 72°C for 30 sec. The melting curve protocol started immediately after amplification. Additionally, the PCR products were run on a 2% agarose gel to confirm their size. The amplification efficiency for each gene and the threshold values for threshold cycle determination (Ct) were determined automatically by the iQ5 Optical System Software (Bio-Rad). Relative mRNA quantification was performed using the ΔCt method for genes with the same amplification efficiency.

### rAAV infection of differentiated hypothalamic neurons culture

After two or ten days in differentiation conditions, hypothalamic neurons were infected with AAV-1/2 vectors expressing the EGFP protein under a neuronal specific promoter, the human Synapsin promoter, previously described [24]. Briefly, half of the culture medium was removed and kept for later. Thereafter, 3.0×10^9^ viral genomes/well of AAV-EGFP were added and eight hours after infection, the remaining half of the culture medium was put back to the well. Seven days after infection, cells were fixed.

### Evaluation of differentiated neurons functionality by single cell calcium imaging

To determine the functional response pattern of differentiated hypothalamic cells, we analyzed the intracellular calcium-free levels ([Ca^2+^]_i_) in single cells following the incubation with ghrelin, leptin or potassium chloride, as previously described with some modifications [38], [39], [40]. Briefly, hypothalamic cells obtained from the differentiation of neurospheres were loaded with 5 µM Fura-2/AM and 0.02% pluronic acid F-127 (both from Molecular Probes) for 45 min at 37°C, in Krebs buffer (pH 7.4) supplemented with 0.1% BSA. After a 10 min post-loading period at room temperature in the Krebs/0.1% BSA, the cover-slip was mounted on an RC-20 chamber in a PH3 platform (Warner Instruments, Hamden, CT). During the experiment, cells were alternately excited at 340 and 380 nm, and a ratio of fluorescence intensity for these wavelengths was obtained (F_340_/F_380_). The fluorescence changes were detected by an Axiocam digital camera coupled to an Axiovert 200 fuorescence microscope (Zeiss) and the image analysis was performed with the Metafluor 2 software (Zeiss). The [Ca^2+^]_i_ changes were analyzed from the cell bodies that were identified as neurons by the procedures reported before [25], [40]. Briefly, the single cells (neurons) are located above the glial cell layer with clear and round nuclei under a phase-contrast microscope and a relatively large diameter (10 µm), and are depolarized by 50 mM KCl. For each neuron analyzed two [Ca^2+^]_i_ values were considered: the basal, corresponds to the value immediately before the stimulus and the peak, corresponds to the maximum value reached by the agent. All agents were dissolved in Krebs buffer. To evaluate the potassium depolarization amplitude, cells were perfused with Krebs buffer and stimulated with KCl 50 mM for 2 minutes. The percentage of cells with significant potassium response (KCl/basal ratio >1.20, corresponding to a 20% increase in [Ca^2+^]_i_) was calculated in two cover-slips (one microscopic field per cover-slip) from independent cell preparations, in a total of approximately 80 cells identified as neurons.

To assess the ghrelin and leptin induced response, cells were perfused with Krebs buffer for 12 minutes and afterwards stimulated with ghrelin (10^−10^ M; Peptide Institute, INC) or leptin (10^−12^ M; Phoenix Pharmaceuticals) for 20 minutes. After a 10 minutes recovery period in Krebs buffer, cells were depolarized with KCl 50 mM for 2 minutes. When the increase in [Ca^2+^]_i_ took place within the 5 minutes after addition of agent and the peak/basal ratio was >1.2 cells were considered responsive. The percentage of responsive cells was calculated in three cover-slips (one microscope field per cover-slip) from independent cell preparations and normalized to the total number of cells identified as neurons.

## Supporting Information

Figure S1 **Morphology of adult hypothalamic neurospheres.** Phase-contrast image of 10–12 DIV hypothalamic neurospheres obtained from adult hypothalamic cells cultured with growth factors in non-adhesive conditions. Scale bar: 20 µm.
(TIFF)

Figure S2 **Adult hypothalamic neurospheres are constituted by neural progenitor cells and express feeding-related neuropeptides NPY, AGRP, POMC and CART.** (A and B) Images of adult hypothalamic neurospheres sections, and (C, D and E) images of adult hypothalamic neurospheres in cover-slips, cultured for 10–12 DIV in proliferative conditions. Adult hypothalamic neurospheres show positive immunostainning for progenitor neural cells marker Musashi-1 (E) and SOX-2 (A, C, D and E) but not for mature neurons marker Neu-N (B). Notice the co-localization of Musashi-1 in SOX-2 positive nuclei (arrows) (E-merge). Adult hypothalamic neurospheres show positive immunostainning for feeding-related neuropeptides NPY (A), AGRP (B), POMC (C) and CART (D). Notice the expression of SOX-2 in NPY-, POMC- and CART- positive cell bodies (arrows) (A-merge, C-merge and D-merge, respectively). DAPI, nuclear staining.
(TIFF)

Supporting Information S1 **Hypothalamic neurospheres from adult rats have progenitor cells and express NPY, AGRP, POMC and CART.**
(DOCX)
