# Supplementary figures and images for: Proliferative Hypothalamic Neurospheres Express NPY, AGRP, POMC, CART and Orexin-A and Differentiate to Functional Neurons
Source: PLoS One. 2011 May 11;6(5):e19745. doi: 10.1371/journal.pone.0019745 (PMC3092771; doi:10.1371/journal.pone.0019745)

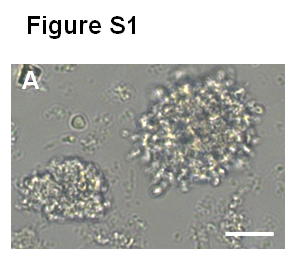

Supplement: Figure S1 — Morphology of adult hypothalamic neurospheres. Phase-contrast image of 10–12 DIV hypothalamic neurospheres obtained from adult hypothalamic cells cultured with growth factors in non-adhesive conditions. Scale bar: 20 µm. (TIFF) [file pone.0019745.s001.tiff]

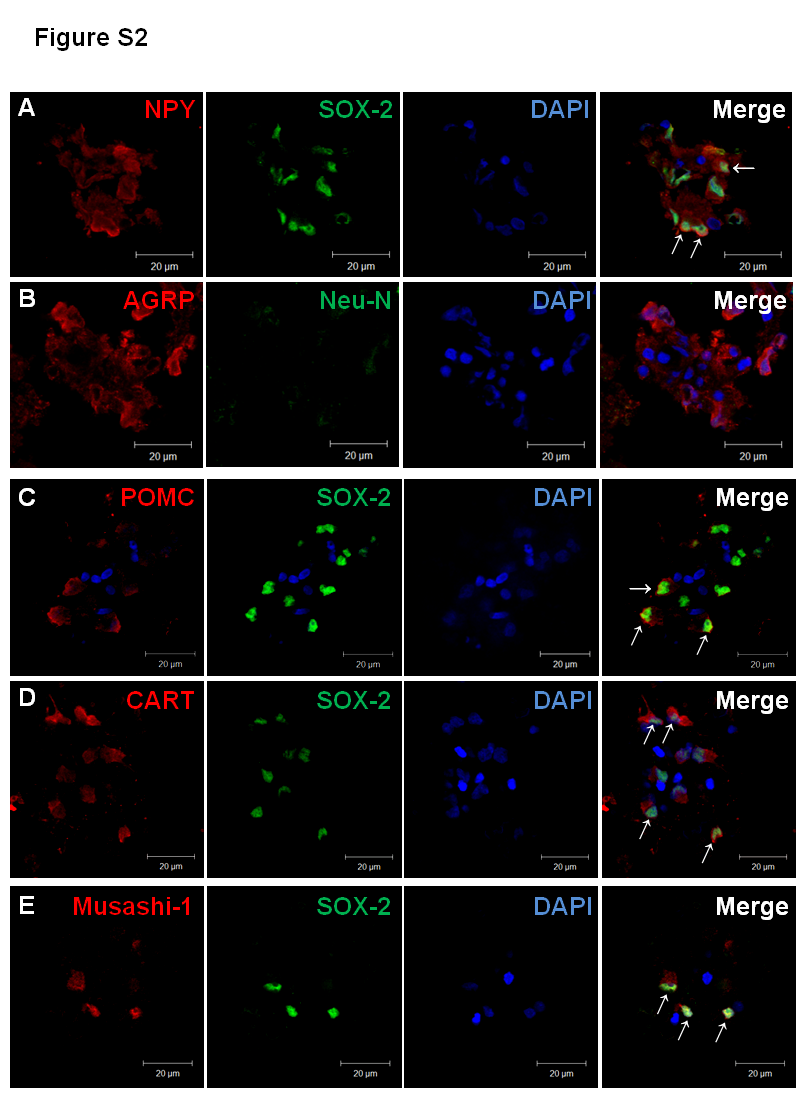

Supplement: Figure S2 — Adult hypothalamic neurospheres are constituted by neural progenitor cells and express feeding-related neuropeptides NPY, AGRP, POMC and CART. (A and B) Images of adult hypothalamic neurospheres sections, and (C, D and E) images of adult hypothalamic neurospheres in cover-slips, cultured for 10–12 DIV in proliferative conditions. Adult hypothalamic neurospheres show positive immunostainning for progenitor neural cells marker Musashi-1 (E) and SOX-2 (A, C, D and E) but not for mature neurons marker Neu-N (B). Notice the co-localization of Musashi-1 in SOX-2 positive nuclei (arrows) (E-merge). Adult hypothalamic neurospheres show positive immunostainning for feeding-related neuropeptides NPY (A), AGRP (B), POMC (C) and CART (D). Notice the expression of SOX-2 in NPY-, POMC- and CART- positive cell bodies (arrows) (A-merge, C-merge and D-merge, respectively). DAPI, nuclear staining. (TIFF) [file pone.0019745.s002.tiff]
