# Supplementary material for: Proliferative Hypothalamic Neurospheres Express NPY, AGRP, POMC, CART and Orexin-A and Differentiate to Functional Neurons
Source: PLoS One. 2011 May 11;6(5):e19745. doi: 10.1371/journal.pone.0019745 (PMC3092771; doi:10.1371/journal.pone.0019745)
Supplement: Supporting Information S1 — Hypothalamic neurospheres from adult rats have progenitor cells and express NPY, AGRP, POMC and CART. (DOCX) [file pone.0019745.s003.docx]

**Supporting Information S1**

**Supporting material and methods**

*Adult hypothalamic neurospheres*

Adult hypothalamic neurospheres were obtained from 8-10 weeks-old Wistar rats purchased from Charles River Laboratories. Briefly, rats were killed by decapitation, the brain was removed and the hypothalamus dissected as described before [1]. Each rat hypothalamus was dissociated and cultured as described in Material and Methods, except for the growth factors which were used at a concentration of 20 ng/mL. Neurospheres were allowed to grow in proliferative conditions for 10-12 days and afterwards collect by centrifugation to O.C.T. compound as described in Material and Methods. The immunocytochemistry was preformed as described for the embryonic hypothalamic neurospheres.

The number of neurospheres obtained from adult hypothalamus is very small, and they are very difficult to identify upon the cryostat. Therefore, after the 10-12 days in culture in proliferative conditions (10-12 DIV), we plated the adult hypothalamic neurospheres and allowed them to adhere to the in Poly-D-coat cover-slips for 18 hours, keeping the same culture medium. In this way, we could identify the neurospheres in the cover-slip more easily and perform the immunocytochemistry as described above.

**Supporting results**

Adult hypothalamic neurospheres are constituted by progenitor cells and express feeding-related neuropeptides NPY, AGRP, POMC and CART

To confirm the presence of progenitor cells in adult hypothalamic neurospheres, we cultured rat hypothalamic cells as floating aggregates in proliferative conditions for 10-12 DIV (Figure S1). Afterwards, we evaluate the expression of progenitor cells markers in adult hypothalamic neurospheres by immunostaining (Figure S2).

Adult hypothalamic neurospheres showed positive immunoreactivity for the multi-potency marker SOX-2 (Figure S2 A, C, D and E) and the neural progenitor marker Musashi-1 (Figure S2 E), but no staining was observed for the mature neurons marker NeuN (Figure S2 E). These data confirm the presence of progenitor undifferentiated cells in the adult hypothalamic neurospheres.

As demonstrate in this study, hypothalamic neurospheres obtained from rat embryonic cells express feeding-related neuropeptides NPY, AGRP, POMC and CART. Therefore, we evaluate whether hypothalamic neurospheres isolated from adult rats express these neuropeptides (Figure S2). In fact, in adult hypothalamic neurospheres showed positive immunoreactivity for NPY, AGRP, POMC and CART (Figure S2 A, B, C and D, respectively). Furthermore, the NPY-, POMC- and CART-cell bodies showed positive immunoreactivity for SOX-2 (arrows) (Figure S2 A-merge, C-merge and D-merge, respectively). These results demonstrate that feeding-related neuropeptides are expressed by adult hypothalamic progenitor cells.

1. Salvi R, Arsenijevic Y, Giacomini M, Rey JP, Voirol MJ, et al. (2009) The fetal hypothalamus has the potential to generate cells with a gonadotropin releasing hormone (GnRH) phenotype. PLoS One 4: e4392.
